# Are Twin Pregnancies at Higher Risk for Iron and Calcium Deficiency than Singleton Pregnancies?

**DOI:** 10.3390/nu15184047

**Published:** 2023-09-18

**Authors:** Anna Dera-Szymanowska, Dorota Filipowicz, Natalia Misan, Krzysztof Szymanowski, Thilo Samson Chillon, Sabrina Asaad, Qian Sun, Ewelina Szczepanek-Parulska, Lutz Schomburg, Marek Ruchała

**Affiliations:** 1 Department of Perinatology and Gynecology, Poznan University of Medical Sciences, Polna 33, 60-535 Poznan, Poland; 2 Department of Endocrinology, Metabolism and Internal Medicine, Poznan University of Medical Sciences, Przybyszewskiego 49, 60-355 Poznan, Poland; 3 Institute for Experimental Endocrinology, Charité-Universitätsmedizin Berlin, D-10115 Berlin, Germany

**Keywords:** calcium homeostasis, iron deficiency, iron deficiency anemia, iron status, iron supplementation, nutrients, trace elements, twin pregnancy

## Abstract

The aim of this study was to compare the iron and calcium status in singleton and twin pregnancies and to assess whether there is an increased risk for iron and calcium deficiency in twin gestation. The study included 105 singleton and 9 twin pregnancies at or above 35 weeks of gestation. Information on prenatal supplementation with iron or calcium was acquired, and adverse perinatal outcomes were recorded. Biosamples from all 114 mothers and 73 newborns (61 singleton and 12 twin newborns) were finally analyzed. Total iron and calcium concentrations in serum were measured through total reflection X-ray fluorescence analysis. The results indicated no significant differences in maternal serum iron and calcium concentrations between singleton and twin pregnancies. Similarly, iron and calcium concentrations in newborn umbilical cord serum samples were not different between singleton and twin pregnancies. The comparison of total iron and calcium between mothers and umbilical cord serum indicated significantly lower concentrations in the mothers, with the differences being not homogenous but rather pair-specific. A significant positive correlation between maternal serum and umbilical cord serum calcium concentration was noticed. Prenatal iron supplementation was associated with higher iron concentrations in both mothers and newborns, supporting the efficiency of supplementation and the quality of the study methods. Collectively, the data indicate no significant differences in serum iron and calcium concentrations with regard to singleton or twin pregnancies and the efficiency of iron supplementation during pregnancy for increasing iron status.

## 1. Introduction

The prevalence of anemia and iron deficiency in the United States is 5.4% and 18.0% in pregnant women, respectively [1]. Both conditions are associated with an increased risk of affected pregnancy course and postpartum period (i.e., impacting thyroid autoimmunity) [2] and adverse perinatal outcomes, such as preterm birth, low birth weight, and low newborn iron stores [3,4,5]. The data suggest that iron plays a major role during fetal brain development, and intrauterine iron insufficiency may increase the risk for motor–cognitive defects in newborns [6,7,8,9]. The changes in cognitive functions were also previously reported in a twin sister with acquired hypothyroidism in comparison to the healthy one, which seems to suggest the possibility of observing twins and their development in the context of microelements deficiencies [10].

The current guidelines recommend the usage of iron-containing supplements during pregnancy, but they do not differentiate between singleton and multiple gestations. Moreover, it is not confirmed if the standard iron content in the typical prenatal supplements (27 mg per dosage) is sufficient for the requirements of high-risk pregnancies. There are only a few reports in the literature concerning iron status and anemia prevalence in healthy women in multiple pregnancies [11,12] or in those who experienced gestational complications or pregnancy-related diseases [13,14,15]. Some expert supplementary guidelines assume higher requirements in twin pregnancies and recommend doubling the iron dosage from 30 to 60 mg of elemental iron daily [16]. However, analytical studies on this issue are scarce, and not all scientific societies agree with this recommendation. The World Health Organization (WHO) [17] and the American College of Obstetricians and Gynecologists (ACOG) [18] do not advise additional iron supplementation in twin pregnancy, whereas the British Society of Hematology (BSH) considers multiple pregnancies to have high risk for iron deficiency. The BSH experts recommend selective iron supplementation in non-anemic women with serum ferritin below 30 µg/L or empirical supplementation with a dose of 40–80 mg of elemental iron daily [19]. These amounts would be compatible with the supplementation recommended by WHO in all pregnant women (30–60 mg) [17].

There is a lack of randomized controlled trials evaluating the proper dosage of iron supplementation in singleton versus twin pregnancies. The expert consensus from the last few decades recommends a decrease in high-dose iron supplementation [20] to a low-dose [17,21] or even a selective one [19,22,23]. Although there are several studies on iron homeostasis in pregnancy, the potentially different requirements for singleton versus twin pregnancies are unclear.

Besides iron, calcium is another important and essential nutrient that is required in increased amounts during pregnancy in order to avoid hypocalcemia and associated pregnancy-related complications [24]. The calcium need of adults is 600 mg/day, which increases up to 1200 mg/day during gestation. The increased requirement for this macroelement ensures proper fetal growth and bone development [25]. The serum calcium concentration decreases in the second and third trimesters of pregnancy, mainly because of hemodilution [26]. Some pregnancy-related complications, such as preeclampsia, low newborn birth weight, preterm delivery, and neonatal death, may be associated with lower serum calcium levels [27,28]. While parathormone and calcitonin do not cross the placenta, calcium is transported actively at a rate of approximately 50 mg/day at 20 weeks of gestation to nearly 330 mg/day at the 35th week of pregnancy [29]. The increased Ca flux results in fetal hypercalcemia, which inhibits parathyroid hormone secretion from the parathyroid glands and stimulates instead the release of calcitonin from the thyroid. With delivery, the placental storage of calcium is no longer available, probably compounded by coexisting hypoparathyroidism and/or hypercalcitonemia that existed in fetal life, causing hypocalcemia. The lowest calcium levels are reached between 24 and 48 h of newborn age before calcium concentration stabilizes again, rising to the levels observed in adults [30].

To address this current knowledge gap, the aim of this study was to characterize the changes in maternal and newborn iron and calcium status in singleton and twin pregnancies. Moreover, the objective of the research was the evaluation of the risk of iron and calcium deficiency in twin gestation. Furthermore, the study considered the influence of prenatal supplementation on iron and calcium concentrations among pregnant women. Finally, the role of maternal serum iron and calcium levels in the prediction of adverse perinatal outcomes was checked. 

## 2. Material and Methods

### 2.1. Study Population

The study was performed in the Department of Perinatology and Gynecology in cooperation with the Department of Endocrinology, Metabolism and Internal Medicine of the Poznan Medical University, and the Institute for Experimental Endocrinology, Berlin Charité Universitätsmedizin, between 2019 and 2021. The study was positively approved by the Bioethics Committee of the Medical University in Poznan (protocol no. 104/19, date of approval: 10 January 2019, annexed 4 February 2021, protocol no. 132/21). The research was performed in compliance with the Helsinki Declaration and fulfilled the appropriate ethics and scientific principles. All women signed an informed consent for the collection and analysis of maternal and umbilical cord blood.

The study included 105 singleton and 9 twin pregnancies at or above 35 weeks of gestation. The material from all 114 mothers and 73 newborns (61 singleton and 12 twin newborns) was finally analyzed. The medical data from the interviews with the patients referred to the obstetric history, the course of actual pregnancy, medications, prenatal supplements, chronic diseases, and perinatal outcomes. All patients underwent a gynecological examination and a fetal ultrasound with Doppler velocimetry (Voluson E10 BT18, GE HealthCare). The estimated fetal weight and its percentile were calculated based on the Hadlock formula [31,32]. 

Women were excluded from the study in any case of confirmed malnutrition, smoking, alcohol consumption, drug intake, cardiovascular or pulmonary disease, hyperparathyroidism, renal failure, Crohn’s disease, ulcerative colitis, malabsorption syndrome, celiac disease, or hemochromatosis. Moreover, the women on any restrictive diet, such as a vegetarian or vegan diet, were also excluded from the study. The pregnancies suspected and confirmed postdelivery to have chromosomal or autosomal abnormalities, uniparental disomy, microdeletions, congenital anomalies, and congenital infections were disqualified from the study. The exclusion criteria also considered the complications of twin pregnancies, such as selective fetal growth restriction, twin to twin transfusion syndrome, or twin anemia-polycythemia syndrome.

### 2.2. Blood Collection

A total amount of 7.5 mL and 2 mL of whole blood was collected from the mother’s peripheral vein and umbilical cord blood, respectively. The blood was collected into Monovette probes without an anticoagulant, stored at room temperature for 30 min to allow coagulation, and then centrifuged at 2700× *g* for 20 min. The serum was carefully removed, placed into sterile Eppendorf tubes, and frozen at −20 °C.

### 2.3. Serum Analysis

The iron and calcium measurements were performed in the analytical laboratory of the Institute for Experimental Endocrinology, Charité-Universitätsmedizin Berlin, by scientists blinded to the clinical information. The iron and calcium levels were measured by total reflection X-ray fluorescence analysis (TXRF spectrometer S4 T-STAR; Bruker Nano GmbH, Berlin, Germany) [33]. The reference ranges for the comparison were derived from the data determined during the analysis of a subsample of the European Prospective Investigation into Cancer and Nutrition (EPIC) study cohort of more than 2000 healthy European adults [34,35]. The reference range for total serum iron, encompassing 95% of values from the reference cohort, was determined to be from 619.8 to 3358.3 μg/L and, for calcium, between 84.6 and 130.5 mg/L. 

### 2.4. Statistical Analysis

The statistical analysis was performed in Statistica StatSoft 13.1 (StatSoft, Cracov, Poland) and PQStat 1.8.2 (PQStatSoftware, Poznan, Poland). The normality of distribution was checked by the use of the Kolmogorov–Smirnov, Lilliefors, and Shapiro–Wilk evaluations. The analysis of unrelated subjects, classified as mothers and newborns, was performed using the non-parametric Mann–Whitney *U* test, while comparisons within mother–child pairs were conducted using the Wilcoxon paired sample test. The data in interval and ordinal scales were described as median (Me), lower quartile (Q1), and upper quartile (Q3). The chi-squared and Fisher’s exact tests were used for analyses on a nominal scale, and the results were presented in percentages (%). The odds ratio (OR) and relative risk (RR) were also calculated. The correlations were evaluated using the Spearman test and described by the use of the correlation coefficient (r). The receiver operating characteristic (ROC) was calculated using DeLong’s method and defined by the area under the curve (AUC), sensitivity, specificity, positive predictive value (PPV), negative predictive value (NPV), and the cut-off. The significance level was set as a *p*-value below 0.05.

## 3. Results

### 3.1. Group Characteristics

The mothers in singleton and twin pregnancies were not different with respect to age. They had comparable values of systolic and diastolic blood pressure. They did not differ according to gravidity and parity. Moreover, no significant differences in the red blood cell parameters and frequency of anemia were observed between the studied groups (Table 1).

### 3.2. Perinatal Outcomes in Singleton and Twin Pregnancies

The mothers in twin pregnancies delivered newborns of significantly lower birth weight and at less advanced gestational weeks; therefore, the twin infants scored lower in all considered anthropometric measurements. Furthermore, the mothers of twin newborns were hospitalized statistically longer than the singleton ones. No significant differences in newborns’ weight on the day of hospital discharge, the 1 and 3 min Apgar score, umbilical cord venous pH, or BE were noticed between the groups (Table 2).

### 3.3. Iron and Calcium Status in Singleton and Twin Pregnancies

**Figure 1:**
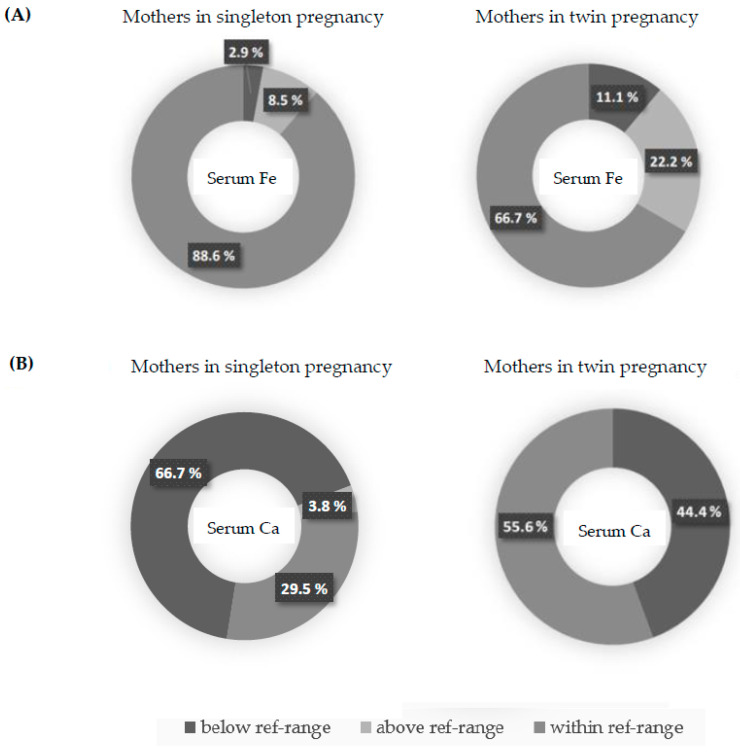
(**A**,**B**) Serum iron (**A**) and calcium (**B**) status among mothers in singleton and twin pregnancy.

**Figure 2:**
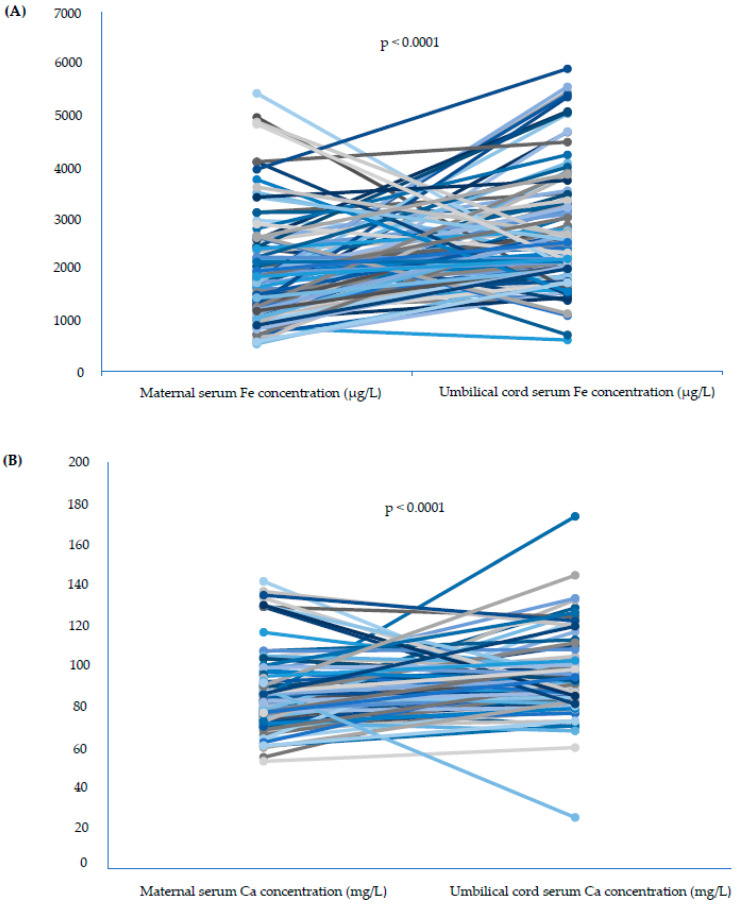
(**A**,**B**) Comparison of serum iron (**A**) and calcium (**B**) concentrations in mother–child pairs.

**Figure 3:**
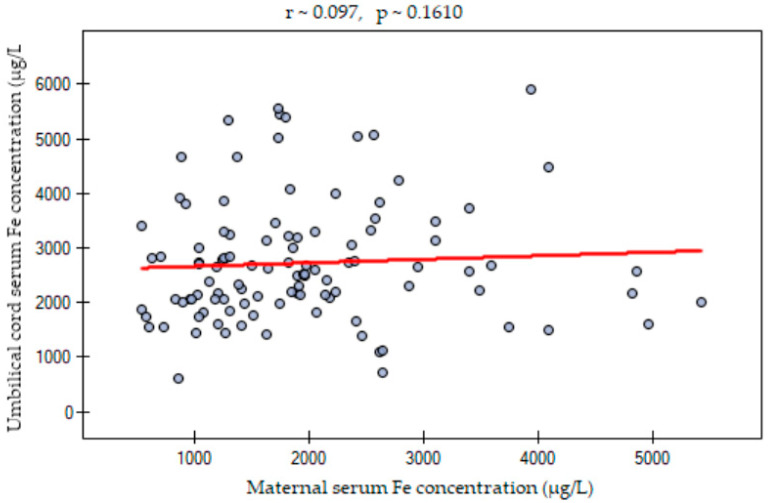
Correlation between maternal and umbilical cord serum iron concentration.

**Figure 4:**
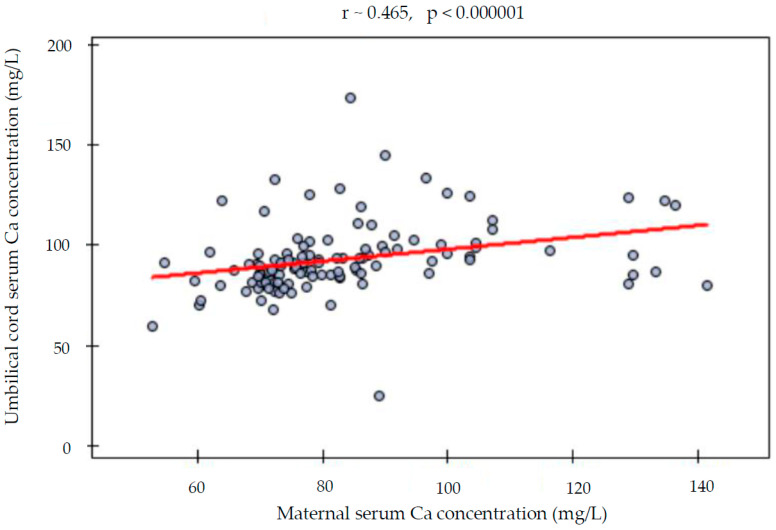
Correlation between maternal and umbilical cord serum calcium concentration.

The maternal serum iron and calcium concentrations were not different between singleton and twin pregnancies. Neither the frequency of iron and calcium normal concentration nor the frequency of deficiency or excess differed significantly between the studied groups (Table 3, Figure 1A,B). There was a statistical trend of higher frequency of proper iron concentrations in singleton pregnancies than among twins, but it was above the significance level. The prevalence of serum hypocalcemia was relatively high both in singleton and twin pregnancies. Also, the OR and RR of inappropriate iron and calcium status were not significantly different (Table 3). Additionally, iron and calcium concentrations of the singleton and twin newborns measured in the umbilical cord serum samples were not statistically different (Table 4). Interestingly, the serum samples from the mothers presented significantly lower iron and calcium concentrations than the umbilical cord blood serum samples (Table 5). A direct analysis between the mothers and their newborns highlighted that the differences were not homogenous but rather pair-specific. Overall, there was a trend of relatively higher iron and calcium concentrations among newborns in comparison to mothers (Figure 2A,B). The association between maternal serum and umbilical cord serum iron levels showed no linear relationship (Figure 3). A significant positive moderate correlation between maternal serum and umbilical cord serum calcium concentration was noticed (Figure 4).

### 3.4. Iron and Calcium Status in Relation to Prenatal Supplementation

The serum iron concentrations were significantly higher among mothers in singleton pregnancies taking prenatal supplementation in comparison to mothers in singleton pregnancies who did not use supplements. This relationship was not observed in twin gestation. No differences in maternal serum calcium levels and umbilical cord serum calcium concentrations in relation to using supplements were found, neither in singleton nor in twin pregnancy (Table 6). Moreover, no significant differences in the maternal serum iron and calcium levels and umbilical cord serum iron and calcium concentrations were observed when comparing both supplemented singleton and twin pregnancies (iron: *p*~0.8360; calcium: *p*~0.7308) and the non-supplemented ones (iron: *p*~0.2460; calcium: *p*~0.5130).

### 3.5. Maternal Serum Iron and Calcium for the Prediction of Adverse Outcomes

**Figure 5:**
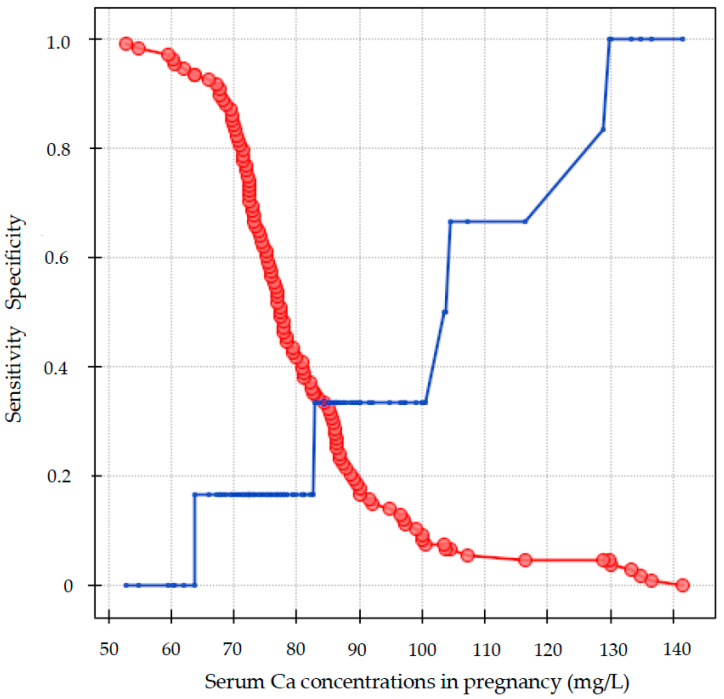
The maternal serum calcium concentration in the prediction of preterm delivery. Red color—sensitivity; Blue color—specificity.

Maternal serum calcium concentrations were associated with preterm delivery. The mothers with gestational serum calcium concentrations below the cut-off value of 63.9 mg/L delivered prematurely with 94% specificity and 95% negative predictive value. The maternal serum iron concentrations were not significantly related to the prognosis of preterm birth and small birth weight. The serum calcium levels in mothers were also unrelated to the prediction of birth weight below 2500 g (Table 7, Figure 5).

## 4. Discussion

This observational study of mothers and newborns from singleton or twin pregnancies describes iron and calcium concentrations in samples from mothers and umbilical cord serum. Active iron supplementation was associated with higher iron serum concentrations. However, no significant differences were observed between the iron and calcium concentrations of singleton or twin pregnancies, neither in the serum samples from the mothers nor in the umbilical cord serum.

### 4.1. The Iron Status in Singleton and Twin Pregnancy

There is a knowledge gap in relation to the requirement of micro- and macronutrients in multiple pregnancies. Only a few studies considered the probability of a higher need for trace element supplementation in twin gestation. Cikim et al. observed significantly lower serum levels of ferritin and zinc concentrations in individuals with single or twin pregnancies compared to healthy non-pregnant women, while the copper levels were statistically higher in pregnancy. Moreover, the iron, ferritin, and zinc levels were decreased among twins as compared with individuals in a single gestation. Furthermore, the researchers observed a non-significant trend towards higher copper concentrations in twin pregnancies [36]. Contrary to the above-cited study, we did not observe significant differences in iron concentrations when comparing mothers or newborns in the singleton or twin pregnancy groups. A reason for this discrepancy may be given in the different populations studied with different dietary patterns and supplies and in the choice of biomarkers for iron status, i.e., serum ferritin versus total serum iron. 

Campbell et al. determined the prevalence of and risk factors for iron deficiency in singleton and twin newborns in a cohort of US American subjects. The frequency of iron deficiency was 21% among twins and 20% among singleton newborns, so it was not different. The serum ferritin levels were related to gestational advancement at delivery, maternal race, and infant sex. The researchers noticed maternal anemia (defined as a hemoglobin level below 11 g/dL) in 40% of cases, but it did not correlate with newborn iron biomarkers, such as serum ferritin, soluble transferrin receptor, or hepcidin [37]. Similarly, our study also observed comparable iron concentrations among singleton and twin newborns. The prevalence of iron deficiency in infants has not been determined because reference standards for total iron in umbilical cord serum are missing.

Shinar et al. evaluated the efficacy of a doubled daily dose of the iron supplement in iron-deficient twin pregnancies. The women were randomized into two groups—the first group received 34 mg of ferrous sulfate, and the second group received twice the amount (68 mg). The study design assumed comparable mean hemoglobin and ferritin levels between groups. Hemoglobin level in the second group was significantly higher from 32 weeks of gestation until 6 weeks postpartum. There were no differences in the secondary outcomes examined. The researchers concluded that a doubled dose of iron increases the hemoglobin and ferritin levels in twin pregnancies [38]. Moreover, the researchers observed that in iron-deficient pregnant women, a single dose of iron was as effective as a double dose [39]. Similarly, Adaji et al. reported that single and double doses of ferrous sulfate have a comparable effect in the prevention of anemia among women who live in areas with a high prevalence of iron deficiency [40]. In our study, we did not notice a difference in iron concentrations regarding prenatal supplementation in twin pregnancies, while the iron levels in supplemented singleton pregnancies were significantly higher than in non-supplemented ones, which may suggest the potential benefits from additional supplementation. The lack of differences in iron levels among mothers of twins can result from the small group size, and further research is required. Moreover, Shinar et al. studied only iron-deficient pregnancies, whereas our study included women with different statuses of red blood cell count at enrollment.

Ru et al. observed that the frequency of tissue iron deficiency (soluble transferrin receptor > 8.5 mg/L) increased significantly across gestation until delivery (9.6% vs. 23.0%). Moreover, the pregnant women with decreased iron storage (serum ferritin < 12 μg/L) were at a two-fold higher risk of anemia at delivery, and 25% of them developed iron deficiency anemia. Overall, 44.6% of women were diagnosed with anemia at delivery, and 18% had iron deficiency anemia. Erythropoietin concentrations during pregnancy were significantly negatively associated with hemoglobin at delivery. Women with erythropoietin > 75th percentile during pregnancy exhibited a three-fold greater risk of anemia, suggesting that erythropoietin is a sensitive predictor of anemia at delivery. The usefulness of ferritin or hepcidin as iron status indicators at delivery is limited because of physiological inflammation processes in the perilabor period [15]. Our study revealed iron deficiency in 2.9% and 11.1% of mothers in singleton and twin pregnancies, respectively, but the groups did not differ significantly. The frequency of anemia in our study was 17.5% in women overall and was more than two times lower than what Ru et al. reported. The serum iron concentrations in newborns were comparable between the groups. The analysis of mother–child pairs showed significantly higher serum iron levels in newborns than in their mothers. Despite that, the umbilical and maternal serum iron concentrations were not associated linearly.

Ru et al. found that umbilical cord hemoglobin or ferritin levels did not change significantly as a function of gestational age at birth (25–38 weeks). Newborns of prenatally obese and smoking women were at a four- to five-fold greater risk of anemia at birth. Moreover, umbilical cord soluble transferrin receptor level was the strongest indicator of umbilical cord hemoglobin, and it correlated significantly with maternal soluble transferrin receptor at mid-gestation and delivery. The umbilical cord iron status was statistically associated with umbilical cord hepcidin but not the maternal one [41]. It indicates the need to perform further studies on iron status and iron metabolism-related factors.

Kosto et al. observed anemia (hemoglobin < 10 g/dL) among 26.7% of twin pregnancies during the second trimester, which is close to our observations. Although the women diagnosed with second trimester anemia were of a higher parity and at a 1.6 times higher risk of blood transfusion, maternal anemia did not increase the risk for adverse perinatal outcomes [12].

Abbass et al. conducted a randomized controlled clinical trial, which included healthy non-anemic women in twin pregnancies at 12 to 16 weeks of gestation. The females were randomized to receive either a single dose (27 mg) or a double dose (54 mg) of elemental iron supplementation. The researchers monitored the hemoglobin concentration at enrollment into the study, at 24 and 32 weeks of gestation, and before delivery. The frequency of iron deficiency anemia was comparable between the single- and the double-dose group (19.1% vs. 24.0%, respectively). In non-anemic participants, hemoglobin concentration differed significantly at fixed time points during gestation. Contrary, Abbass et al. did not find a significant difference in the hemoglobin levels between the studied groups, and the researchers concluded that the doubled prophylactic iron dosage gives no benefits in twin pregnancies not diagnosed with anemia. The researchers consider the iron requirements in twin pregnancy to be increased by 1.8 times in comparison to singletons, secondarily to the relatively higher feto-placental need and greater expansion in maternal plasma volume and erythrocyte mass. There are only a few reports considering if the standard antenatal iron supplementation is sufficient for higher iron requirements in iron deficiency prevention among twins [23]. The study of Abbass et al. enforces the need for further research in a larger group of patients who would have been diagnosed at enrollment to the study or at other pregnancy time points with iron deficiency anemia.

Ali et al. randomly assigned non-anemic women in twin pregnancy into two groups receiving 27 mg or 54 mg daily of elemental iron supplementation between 12 and 36 weeks of gestation. The researchers reported that both iron dosages ensured that the mean hemoglobin and hematocrit levels were within the normal range. The mean serum ferritin concentrations were significantly higher in the doubled-iron-dose group at 36 weeks of gestation. Furthermore, women from the second group more often reported side effects [42]. The observations of Ali et al. and Abbass et al. are in line with our results. Although the maternal serum iron concentrations were significantly lower among women in singleton pregnancies, who did not use prenatal supplementation in our study, this difference was at the verge of statistical significance. Moreover, we did not notice a significant difference in the maternal serum iron level when comparing the supplemented twin and singleton pregnancies.

### 4.2. The Calcium Status in Singleton and Twin Pregnancy

Kant et al. reported the prevalence of hypocalcemia in 23.9% of pregnant women in North India. The mean serum calcium level was 9.56 mg/dL, which was within the normal range for pregnant women [24]. The frequency of decreased serum calcium concentration in our study was higher than that noticed by Kant et al. and was found to be 66.7% and 44.4% of women in singleton and twin pregnancy, respectively, but the difference was not significant. The umbilical cord serum calcium level was comparable between singleton and twin newborns in our study but was significantly higher among newborns in comparison to their mothers. Although this relationship was not homogenous, it stayed significant in direct comparisons between mothers and newborns; however, it was stronger when analyzed in mother–child pairs. Kant et al. observed that serum calcium concentration did not correlate with dietary calcium intake. Mean serum calcium level was statistically lower in mothers who delivered low birth weight newborns when compared to mothers who had appropriate birth weight infants. Serum calcium concentrations were not associated with preeclampsia, preterm delivery, or neonatal mortality [24]. In contrast, other studies reported that serum calcium levels were lower in preeclamptic women as compared to physiological gestation [43,44,45]. An et al. confirmed in a meta-analysis that there is no effect of calcium supplementation on preeclampsia [46]. Our study did not consider the association between serum calcium levels and preeclampsia because the patients were not diagnosed with hypertension disorders. Moreover, it has been observed that the serum calcium concentration may be useful in the prediction of preterm delivery, but it should be emphasized that this relationship was considered independent of the magnesium status. Grzeszczak et al. studied multiple pregnancies and observed a strong significant positive correlation between calcium and magnesium concentrations in the umbilical and the fetal membrane as well as between umbilical cord magnesium concentrations and the length of the pregnancy [47]. 

Nakayama et al. studied bone formation and resorption markers, mineral metabolism, and calcium-regulating factors in singleton and twin pregnancies. The assessed parameters were evaluated at 10, 25, 30, and 36 weeks of gestation and at 4 days and 1 month postpartum. The researchers reported significantly higher urinary levels of cross-linked type I collagen N-telopeptides and C-terminal telopeptides of type I collagen among females in twin pregnancy when compared to women in singleton gestation. Moreover, the high levels of the abovementioned parameters were noticed earlier in twin than in singleton pregnancies. Furthermore, serum concentrations of bone-specific alkaline phosphatase, calcium, and phosphate were significantly higher in twin gestation than in singleton pregnancy, while the 1,25-(OH)2 vitamin D and 25-(OH) vitamin D levels were lower [48]. Similarly, we observed a trend towards higher serum calcium concentrations in twin pregnancy as compared to singleton gestation, but it was above the significance level. 

Contrary to the above-cited study and our observations, Goswami et al. observed significantly lower serum calcium concentrations among twins when compared to singleton newborns delivered above 28 weeks of gestation in an observational study conducted in India. The researchers reported maternal 25-(OH) vitamin D deficiency in 90% of twin and 88% of singleton pregnancies. The frequency of newborn vitamin D insufficiency was 89% in twin and 74% in singleton gestation. Maternal serum 25-(OH) vitamin D concentration was significantly lower in twin compared to singleton gestations. Mean maternal serum calcium level did not differ between the groups, in accordance with our study. Moreover, maternal and newborn 25-(OH) vitamin D levels were positively associated both in singleton and twin pregnancies [49]. Our study revealed a positive correlation between maternal and umbilical cord serum calcium concentration, but all mothers and newborns were considered together.

Haliloglu et al. observed no significant differences in serum calcium levels among pregnant women in Turkey, measured in the 12th, 25th, and 32nd gestational weeks and 6 weeks postpartum, compared to the healthy non-pregnant females. Moreover, the researchers noticed no statistical differences in serum calcium levels between the second and third trimesters of pregnancy. Considering the other parameters of calcium metabolism, Haliloglu et al. reported a 13.3% and 33.3% deficiency rate of 25-(OH) vitamin D in the 32nd week and the postpartum period [50]. In view of all these partly conflicting results, there is a need to examine the calcium metabolism and the calcium-regulating factors in pregnancy in larger and more homogenous groups of twins, also in the context of baseline micronutrient and macronutrient supply that differs grossly between populations residing in different geographical areas and between women with different dietary patterns, such as unrestricted versus vegetarian or vegan diets and taking or not taking the recommended supplemental intake of essential vitamins, minerals, and trace elements.

### 4.3. The Strengths and Limitations

The strength of the study is the analysis of the iron and calcium concentrations not only in individuals but also in mother–child pairs. Moreover, this is one of the few studies that considers iron and calcium homeostasis in twin pregnancy, also in the context of the need for supplemental intake of this nutrient. 

The limitation of the study is the enrollment of a relatively small group of mothers in twin pregnancy, not allowing a separate assessment of monochorionic and dichorionic twins separately. Moreover, we evaluated the iron and calcium concentrations in the form of total serum concentrations and did not assess the other biomarkers of iron and calcium status. Future studies on iron and calcium homeostasis-related parameters, such as ferritin, hepcidin, vitamin D, parathormone, and calcitonin, are needed in relation to the total serum concentrations to determine the strength of correlation between the biomarkers and to decide on the most informative parameters for detecting deficiency and additional requirements in pregnancy.

## 5. Conclusions

This observational study of singleton and twin pregnancies indicated that Polish women are at some risk of iron and calcium deficiency. The prevalence of low iron and calcium status was not statistically different between singleton and twin pregnancies, and supplemental iron intake proved effective in increasing the iron status of pregnant women, as evidenced in the group of singleton pregnancies. Interestingly, there was no linear correlation between the iron and calcium status of mothers with the concentrations in umbilical cord serum, highlighting that the transfer across the placenta is actively controlled and not dominated by random diffusion. Further research on the factors affecting iron and calcium transfer during pregnancy for meeting the requirements of twin versus singleton pregnancies is needed with larger cohorts of women and taking geographical conditions and the baseline supply into consideration. A better database on this issue will improve pregnancy care and help in identifying relevant risk factors and specific requirements for the mothers and for successful and regular delivery.

## Figures and Tables

**Table 1:** The group characteristics.

	Mothers in Singleton Pregnancy (*n* = 105)	Mothers in Twin Pregnancy (*n* = 9)	*p*-Value
	Me (Q1–Q3)	Me (Q1–Q3)	
Age (years)	34 (32–36)	34 (32–36)	0.9297
SBP (mm Hg)	110 (100–120)	118 (111–120)	1.0000
DBP (mm Hg)	70 (60–73)	65 (60–73)	1.0000
Gravidity (n)	2 (1–2)	2 (2–2)	0.4053
Parity (n)	0 (0–1)	1 (0–1)	0.2729
RBC (T/L)	4.19 (3.9–4.4)	4.4 (4.12–4.59)	0.2269
Hb (mmol/L)	7.6 (6.9–8.0)	7.2 (7.0–7.7)	0.7093
Ht (L/L)	0.363 (0.334–0.381)	0.355 (0.345–0.375)	0.9382
MCV (fL)	87.6 (84.6–90.0)	85.2 (85.0–86.2)	0.1029
MCH (fmol)	1.84 (1.74–1.91)	1.75 (1.7–1.79)	0.0771
MCHC (mmol/L)	20.9 (20.5–21.2)	20.3 (19.9–20.5)	0.0814
RDW (%)	13.2 (12.7–13.6)	13.1 (12.9–13.7)	0.8970
Prevalence of anemia (%) *	17.1	22.2	0.6565

SBP—systolic blood pressure, DBP—diastolic blood pressure, RBC—red blood cells, Hb—hemoglobin, Ht—hematocrit, MCV—mean cell volume, MCH—mean cell hemoglobin, MCHC—mean cell hemoglobin concentration, RDW—red cell distribution width, * hemoglobin concentrations below 10.5 g/dL in the IInd/IIIrd trimester of pregnancy—it refers to the British Committee for Standards in Haematology recommendations [19].

**Table 2:** Perinatal outcomes in singleton and twin pregnancies.

	Singleton Pregnancy (*n* = 105)	Twin Pregnancy (*n* = 9)	*p*-Value
	Me (Q1–Q3)	Me (Q1–Q3)	
The week of delivery	39 (38–40)	36 (36–37)	**0.0001**
Birth weight (g)	3475 (3180–3760)	2740 (2380–3020)	**0.0001**
The newborn weight at the day of hospital discharge (g)	62 (56–70)	66 (62–79)	0.1136
1 min Apgar score (points)	10 (10–10)	10 (9–10)	0.4603
3 min Apgar score (points)	10 (10–10)	10 (10–10)	0.2542
Umbilical venous pH	7.28 (7.22–7.33)	7.31 (7.26–7.33)	0.5037
Umbilical venous BE	−2.57 (−5.38–−0.52)	−2.31 (−4.31–−1.68)	0.9958
Body length (cm)	54 (53–56)	50 (49–52)	**0.0001**
Head circumference (cm)	34.5 (33.5–35.5)	33 (32–35)	**0.0268**
Thoracic circumference (cm)	34 (33–35)	31 (30–32)	**0.0001**
The length of hospitalization (days)	5 (4–6)	6 (6–9)	**0.0186**

Me—median, Q1—first quartile, Q3—third quartile, *p*—the level of significance for the Mann–Whitney *U* test, pH—potential of hydrogen, BE—base excess. The bold means that the result is statistically significant.

**Table 3:** The iron and calcium status among mothers in singleton and twin pregnancies.

	Mothers in Singleton Pregnancy (*n* = 105)	Mothers in Twin Pregnancy (*n* = 9)	*p*-Value	OR	*p*-Value	RR	*p*-Value
	Me (Q1–Q3)	Me (Q1–Q3)					
Serum Fe concentration (µg/L)	1729.5 (1204.7–2346.0)	2048.0 (1175.6–3098.0)	0.6273	-	-	-	-
below ref-range (%)	2.9	11.1	0.2836	4.25 (0.39–45.68)	0.2324	3.88 (0.44–33.66)	0.2174
within ref-range (%)	88.6	66.7	0.0955	0.25 (0.05–1.16)	0.0788	0.75 (0.47–1.20)	0.2331
above ref-range (%)	8.5	22.2	0.2093	3.04 (0.54–16.91)	0.2024	2.59 (0.65–10.22)	0.1737
Serum Ca concentration (mg/L)	77.5 (72.4–86.4)	103.5 (70.2–107.2)	0.1491	-	-	-	-
below ref-range (%)	66.7	44.4	0.2739	0.40 (0.10–1.58)	0.1918	0.66 (0.31–1.40)	0.2847
within ref-range (%)	29.5	55.6	0.1382	2.98 (0.75–11.86)	0.1205	1.88 (0.97–3.62)	0.0584
above ref-range (%)	3.8	0.0	1.0000	1.18 (0.05–23.76)	0.9106	1.17 (0.06–20.33)	0.9103

Fe—iron, Ca—calcium, Me—median, Q1—first quartile, Q3—third quartile; *p*—level of significance for Mann–Whitney *U* test, OR—odds ratio, RR—relative risk.

**Table 4:** The iron and calcium status among newborns from singleton and twin pregnancies.

	Newborns from Singleton Pregnancy (*n* = 61)	Newborns from Twin Pregnancy (*n* = 12)	*p*-Value
	Me (Q1–Q3)	Me (Q1–Q3)	
Umbilical cord serum Fe concentration (µg/L)	2514.0 (2021.5–3283.0)	2938 (2283.0–3451.0)	0.2431
Umbilical cord serum Ca concentration (mg/L)	90.4 (82.4–97.8)	93.5 (84.4–100.5)	0.4717

Fe—iron, Ca—calcium, Me—median, Q1—first quartile, Q3—third quartile; *p*—level of significance for Mann–Whitney *U* test.

**Table 5:** The comparison of iron and calcium status between mothers and newborns.

	Mothers (*n* = 114)	Newborns (*n* = 73)	*p*-Value for Mann–Whitney *U* Test	*p*-Value for Wilcoxon Test
	Me (Q1–Q3)	Me (Q1–Q3)		
Serum Fe concentration (µg/L)	1745.6 (1222.5–2398.0)	2562.0 (2031.0–3231.0)	**0.0001**	**<0.0001**
Serum Ca concentration (mg/L)	78.1 (72.1–89.1)	90.8 (83.3–98.4)	**0.0001**	**<0.0001**

Fe—iron, Ca—calcium, Me—median, Q1—first quartile, Q3—third quartile; *p*—level of significance for Mann–Whitney *U* test. The bold means that the result is statistically significant.

**Table 6:** The iron and calcium status in singleton and twin pregnancies in relation to taking prenatal supplements.

	Singleton Pregnancies (*n* = 103)			Twin Pregnancies (*n* = 9)		
	Prenatal Supplementation (+) (*n* = 67)	Prenatal Supplementation (−) (*n* = 36)	*p*-Value	Prenatal Supplementation (+) (*n* = 5)	Prenatal Supplementation (−) (*n* = 4)	*p*-Value
	Me (Q1–Q3)	Me (Q1–Q3)		Me (Q1–Q3)	Me (Q1–Q3)	
Maternal serum Fe concentration (µg/L)	1817.6 (1257.2–2386.5)	1518.7 (944.0–1901.7)	**0.0464**	2048.0 (1030.8–2222.0)	2352.0 (1274.9–3568.5)	1.0000
Maternal serum Ca concentration (mg/L)	79.3 (73.1–86.9)	76.2 (71.6–84.6)	0.2403	82.8 (70.2–104.6)	116.2 (95.1–129.1)	1.0000
Umbilical cord blood serum Fe concentration (µg/L)	2512.0 (2058.0–3220.0)	2382.0 (1731.0–2826.0)	0.2057	2938.0 (2611.0–3372.5)	2562.0 (1952.4–3483.0)	0.5361
Umbilical cord blood serum Ca concentration (mg/L)	91.1 (82.4–99.9)	87.7 (82.8–95.2)	0.3569	97.2 (83.7–106.2)	91.3 (85.7–94.4)	0.6330

Fe—iron, Ca—calcium, Me—median, Q1—first quartile, Q3—third quartile; *p*—level of significance for Mann–Whitney U test. The bold means that the result is statistically significant.

**Table 7:** Maternal serum iron and calcium concentrations in the prediction of adverse perinatal outcomes.

	AUC	Cut-Off	Sensitivity (%)	Specificity (%)	PPV (%)	NPV (%)	*p*-Value
Maternal serum Fe concentrations (μg/L)							
Preterm birth	0.490	<1175.6	50	79	12	97	0.9082
Birth weight < 2500 g	0.685	<965.2	67	88	24	98	0.1275
Maternal serum Ca concentrations (mg/L)							
Preterm birth	0.253	<63.9	17	94	13	95	**0.0423**
Small birth weight	0.469	<71.3	50	81	13	97	0.7996

Fe—iron, Ca—calcium, AUC—area under curve, cut-off—cut-off value, PPV—positive predictive value, NPV—negative predictive value, *p*–the level of significance for the Mann–Whitney *U* test.

## Data Availability

The data are accessible upon reasonable request to the senior author.
